# Glucose-regulated protein 58 modulates β-catenin protein stability in a cervical adenocarcinoma cell line

**DOI:** 10.1186/1471-2407-14-555

**Published:** 2014-08-01

**Authors:** Chia-Jung Liao, Tzu-I Wu, Ya-Hui Huang, Ting-Chang Chang, Chyong-Huey Lai, Shih-Ming Jung, Chuen Hsueh, Kwang-Huei Lin

**Affiliations:** Department of Biochemistry, Chang-Gung University, 259 Wen-hwa 1 Road, Taoyuan, 333 Taiwan; Department of Obstetrics and Gynecology, Wan Fang Hospital, Taipei Medical University, Taipei, 116 Taiwan; Medical Research Center, Chang Gung Memorial Hospital, Taoyuan, 333 Republic of China; Department of Obstetrics and Gynecology, Chang Gung Memorial Hospital, Taoyuan, Taiwan, 333 Taiwan; Department of Pathology, Chang Gung Memorial Hospital, Taoyuan, 333 Taiwan; Pathology Core of Chang Gung Molecular Medicine Research Center, Chang-Gung University, Taoyuan, 333 Taiwan

## Abstract

**Background:**

Cervical cancer continues to threaten women’s health worldwide, and the incidence of cervical adenocarcinoma (AD) is rising in the developed countries. Previously, we showed that glucose-regulated protein 58 (Grp58) served as an independent factor predictive of poor prognosis of patients with cervical AD. However, the molecular mechanism underlying the involvement of Grp58 in cervical carcinogenesis is currently unknown.

**Methods:**

DNA microarray and enrichment analysis were used to identify the pathways disrupted by knockdown of Grp58 expression.

**Results:**

Among the pathway identified, the WNT signaling pathway was one of those that were significantly associated with knockdown of Grp58 expression in HeLa cells. Our experiments showed that β-catenin, a critical effector of WNT signaling, was stabilized thereby accumulated in stable Grp58 knockdown cells. Membrane localization of β-catenin was observed in Grp58 knockdown, but not control cells. Using a transwell assay, we found that accumulated β-catenin induced by Grp58 knockdown or lithium chloride treatment inhibited the migration ability of HeLa cells. Furthermore, an inverse expression pattern of Grp58 and β-catenin was observed in cervical tissues.

**Conclusions:**

Our results demonstrate that β-catenin stability is negatively regulated by Grp58 in HeLa cells. Overexpression of Grp58 may be responsible for the loss of or decrease in membranous β-catenin expression in cervical AD.

**Electronic supplementary material:**

The online version of this article (doi:10.1186/1471-2407-14-555) contains supplementary material, which is available to authorized users.

## Background

Cervical cancer is the third leading cause of cancer-related mortality among women worldwide [1], although records show a marked decline in incidence over the past three decades. Despite a reducing in the incidence of cervical squamous cell carcinoma (SCC), the frequency of cervical adenocarcinoma (AD) is increasing due to insufficient detection of cervical AD precursor lesions with the Papanicolaou smear test [2]. Therefore, identification of biomarkers specific for cervical AD is essential for early detection and improved prognosis. Persistent infection with high-risk human papillomavirus (HPV) is the major risk factor for both SCC and AD [3]. However, HPV alone is not sufficient to cause cervical cancer; other molecular markers of cervical carcinogenesis are essential. Previously, we demonstrated that glucose-regulated protein 58 (Grp58) serves as an independent prognostic factor for cervical AD, but not SCC [4]. Cell-based studies revealed that Grp58 regulates the invasion and metastatic ability of HeLa cells. Grp58 is a multi-functional protein belonging to the disulfide isomerase family of proteins [5]. The functions of Grp58 in quality control of glycoprotein and major histocompatibility complex class I (MHC class I) maturation are well documented [6]. Recent evidence has suggested that Grp58 plays a role in cancers [7, 8], although the details are unclear. In the current investigation, we explored the role of Grp58 in cervical AD progression and the molecular mechanism underlying Grp58 function.

## Methods

### Pathway enrichment analysis

Pathway enrichment analysis of a set of differentially expressed genes upon Grp58 knockdown was performed using the GeneGo MetaCore analysis tool (GeneGo, St. Joseph, MI). Genes displaying differential expression, by comparing stable control and Grp58 knockdown cells, greater than 1.2 fold were uploaded. A pathway map with a false discovery rate of <0.01 was considered significant.

### Cell lines and cultures

The human cervical cancer cell line, HeLa, was obtained from the American Type Culture Collection (ATCC, Number: CCL-2), and cultured as recommended. Stable Grp58 knockdown cells were established as described earlier [4]. For MG132 (Sigma-Aldrich, St. Louis, MO) and LiCl (Sigma-Aldrich) treatment, cells were seeded and incubated overnight. The culture medium was refreshed, and MG132 (10 μM) or LiCl (20 or 40 mM) was added to the culture medium at 4 and 24 h prior to harvest, respectively. For the Boyden chamber assay, LiCl was added to the upper and lower chambers during cell seeding. For analysis of β-catenin degradation, cells were pre-treated with MG132 for 4 h. The medium was refreshed and cycloheximide (CHX, 10 ng/ml; Sigma-Aldrich) was used to block new protein synthesis. Cells were harvested at 0, 1, 2, and 4 h after treatment with CHX.

### Real-time quantitative RT-PCR (qRT-PCR)

Total RNA was extracted from cells using TRIzol. The first cDNA strand was synthesized using the superscript III kit for RT-PCR (Life Technologies, USA). qRT-PCR was performed using SYBR Green, as described previously [9]. The primer sequences for β-catenin were as follows: forward, 5’-CCg CAA ATC ATg CAC CTT T-3’, and reverse, 5’-CTg ATg TgC ACg AAC AAg CA-3’. Primers used in supplementary data were listed in Additional file 1: Supplementary Methods and Figures.

### Western blot analysis

Western blot analysis was performed as described previously [10]. Anti-Grp58 rabbit polyclonal antibody (1:10,000 dilution; Atlas, Sigma-Aldrich, St. Louis, MO), anti-β-catenin mouse monoclonal antibody (1:2000 dilution; E-5 clone; Santa Cruz Biotechnology Inc., Santa Cruz, CA) and horseradish peroxidase-conjugated, affinity-purified secondary antibody to rabbit or mouse (Santa Cruz Biotechnology) were used. Immunocomplexes were visualized via chemiluminescence with an ECL detection kit (Amersham, Piscataway, NJ).

### Transwell assay

Cells were trypsinized and re-suspended using serum-free medium. Equal amounts of cells (5×10^4^ in 100 μl) were seeded in the upper chamber (Corning-Costar 3494 Transwell, Lowell, MA) in triplicate. Lower chambers were supplemented with 20% fetal bovine serum in medium. Traversed cells were stained with crystal violet after 24 h incubation.

### Immunofluorescence (IF) staining

Cells were seeded on glass slides, fixed with 3.7% paraformaldehyde, permeabilized with 0.1% Triton X-100/PBS (PBST) for 10 min, blocked with 1% bovine serum albumin for 30 min, and stained with the indicated primary antibody for 3 h at RT. After washing three times with PBST, slides were incubated with secondary antibody for 2 h at RT. Fluorescence images were acquired using confocal microscopy (ZEISS LSM 510 META, Carl Zeiss Inc., Oberkochen, Germany). Grp58 and β-catenin primary antibodies were the same as those used for Western blotting. The secondary antibodies employed were Alexa Fluor 488 goat-anti-mouse and 568 goat-anti-rabbit antibody (Invitrogen Co., Carlsbad CA).

### Immunohistochemistry (IHC) staining

Formalin-fixed and paraffin-embedded tissues were examined using IHC, according to previously described procedures [11]. The Grp58 (Atlas, 1:2000 dilution) and β-catenin (Santa Cruz Biotechnology, Inc., Santa Cruz, CA; 1:100 dilution) antibodies were used, along with horseradish peroxidase-conjugated anti-rabbit and anti-mouse secondary antibodies (Santa Cruz). Immunocomplexes were visualized using the Envision kit (DAKO, Carpinteria, CA). Brown-colored cytoplasmic patches were considered Grp58-positive. Slides were scored separately by two independent pathologists (Y.L and S.M.J) blinded to all clinical data. Staining intensity was graded as absent (0), weak (1+), medium (2+) or strong (3+). The histoscore (Q) was calculated by multiplying the percentage (P) of positive cells by intensity (I), according to the formula: Q = P × I. The mean Q of each cervical cancer type was selected as the cut-off value to divide the high/low expression groups, as described previously.

### Study population

Data from a total of 109 cervical carcinoma patients subjected to primary definitive surgery between 2000 and 2008 at Chang Gung Memorial Hospital (Taoyuan, Taiwan) were retrieved from the hospital database, and the histological types confirmed by pathologists. Thirty-four patients with cervical AD, classified as stage I to IIB according to the International Federation of Gynecology and Obstetrics (FIGO) staging system, were enrolled under the protocol approved by the Institutional Review Board (IRB: 95-1241B); all patients provided informed consent. The personal rights of the patients were preserved.

### Statistical analysis

One-way analysis of variance (ANOVA) was used to compare means of more than two groups. Mann–Whitney test was applied to compare the means of two independent groups. *P* values < 0.05 were considered significant. SPSS statistical software was used for statistical analyses.

## Results

### Altered WNT signaling pathway in Grp58-knockdown HeLa cells

Previously, we used an Affymetrix microarray to identify the genes disrupted upon knockdown of Grp58 expression [4]. Differentially expressed genes were assessed with the MetaCore pathway analysis tool. A total of 1218 Affymetrix probe IDs (fold change > 1.2 and <0.8) were imported, among which 1208 were identified. Eighteen pathway maps were significantly enriched based on the differentially expressed genes (false discovery rate < 0.01), which are listed in Table 1. Intriguingly, three WNT-related maps (listed in 2, 15 and 16 of Table 1) were enriched. The Wnt signaling pathway, originally discovered in Drosophila, is highly conserved among species. Wnt signaling regulates diverse processes during embryo development, maintenance of tissue homeostasis, and pathological conditions, including cancer [12]. Several WNT signaling pathway-related genes, including frizzled drosophila homolog of 10, disheveled homolog 1, casein kinase I epsilon and casein kinase II alpha chain, and WNT signaling target genes, including Myc, CD44, lymphoid enhancer-binding factor 1, and vimentin, were disrupted in Grp58 knockdown cells [4]. The mRNA and protein levels of several differentially expressed genes were verified using real-time quantitative RT-PCR (qRT-PCR) and Western blot analysis, respectively (Additional file 2: Figure S1A, B).

**Table 1:** **Pathway map enrichment analysis**

#	Maps	Maps folder	p value
1	Granzyme A signaling	Apoptosis and survival	6.81E-08
2	TGF, WNT and cytoskeletal remodeling	Cytoskeleton remodeling	2.68E-07
3	Antigen presentation by MHC class I	Immune response	3.65E-07
4	IGF-1 receptor signaling	Development	4.78E-06
5	Androgen Receptor nuclear signaling	Transcription	6.57E-06
6	Glucocorticoid receptor signaling	Development	1.01E-05
7	ATM/ATR regulation of G1/S checkpoint	DNA damage	1.34E-05
8	LRRK2 in neurons in Parkinson's disease		1.77E-05
9	GM-CSF signaling	Development	1.94E-05
10	Endothelial cell contacts by junctional mechanisms	Cell adhesion	1.98E-05
11	Cytoskeleton remodeling	Cytoskeleton remodeling	2.64E-05
12	AKT signaling	Signal transduction	2.78E-05
13	Endoplasmic reticulum stress response pathway	Apoptosis and survival	3.48E-05
14	Role of Activin A in cell differentiation and proliferation	Development	9.27E-05
15	WNT signaling pathway. Part 2	Development	1.84E-04
16	WNT signaling pathway. Part 1. Degradation of beta-catenin in the absence WNT signaling	Development	1.92E-04
17	TGF-beta-dependent induction of EMT via SMADs	Development	2.03E-04
18	Mismatch repair	DNA damage	2.63E-04

### Grp58 regulates β-catenin protein stability

**Figure 1:**
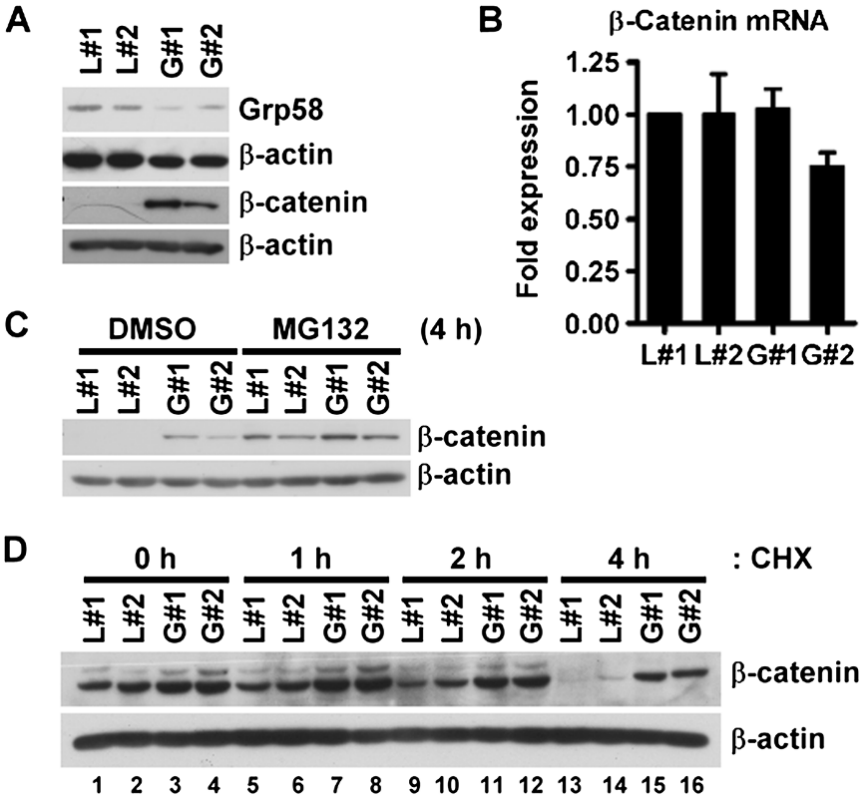
**β-catenin protein is accumulated in Grp58 knockdown cells.** Grp58 and β-catenin protein levels in stable Grp58 knockdown cells were determined using Western blot **(A, C and D)** and qRT-PCR **(B)**. **(C)** Cells were pretreated with vehicle control (DMSO) or MG132 for 4 h before harvest. **(D)** β-Catenin degradation assay. After pre-treatment with MG132 for 4 h, the medium was refreshed. Cells were subsequently treated with CHX and harvested at the indicated time-points. L#1 and L#2, HeLa cell lines stably expressing luciferase shRNA; G#1 and G#2, HeLa cell lines stably expressing Grp58 shRNA.

β-Catenin is a crucial molecule of the WNT signaling pathway. The β-catenin-dependent canonical pathway is the Wnt signaling pathway that has been extensively characterized to date. β-catenin transduces Wnt-activated signals to the nucleus and serves as a transcriptional co-activator in T cell factor/lymphoid enhancer-binding factor (TCF/LEF)-dependent gene transcription [13]. Several β-catenin target genes, such as L1 cell adhesion molecule (L1CAM) [14], laminin β-3 (LAMB3) [15], LEF1 [16], Myc [17] and S100A4 [18] were identified in our DNA microarray analysis. In view of this finding, it is proposed that the Wnt-β-catenin canonical pathway is regulated upon knockdown of Grp58 expression. Consistent with this theory, increased β-catenin-TCF transactivation activity was observed in stable Grp58 knockdown cells (G#1 and G#2, Additional file 2: Figure S1C). Accordingly, the β-catenin mRNA and protein levels in Grp58 knockdown cells were examined. As shown in Figure 1A, β-catenin protein was more abundant in stable Grp58 knockdown cells (G#1 and G#2), compared to control cells (L#1 and L#2). However, no significant differences in β-catenin mRNA levels were observed between the cell groups (Figure 1B), explaining why β-catenin was not identified in microarray analysis. We propose that Grp58 regulates β-catenin expression via modulating protein stability, but not gene transcription. β-Catenin protein expression was indistinguishable in control and knockdown cells treated with MG132, a proteasome inhibitor (Figure 1C). The half-life of β-catenin was subsequently analyzed. CHX was used to block new synthesis of protein. Notably, the β-catenin protein was stabilized and accumulated in Grp58 knockdown cells after 4 h of CHX treatment (Figure 1D, lanes 15 and 16). In contrast, traces of β-catenin were barely detectable in control cells at this time-point (Figure 1D, lanes 13 and 14). The data suggest that Grp58 regulates β-catenin protein degradation, but not gene transcription, in HeLa cells.

### β-catenin accumulates in cell-cell adherens of Grp58-knockdown HeLa cells

**Figure 2:**
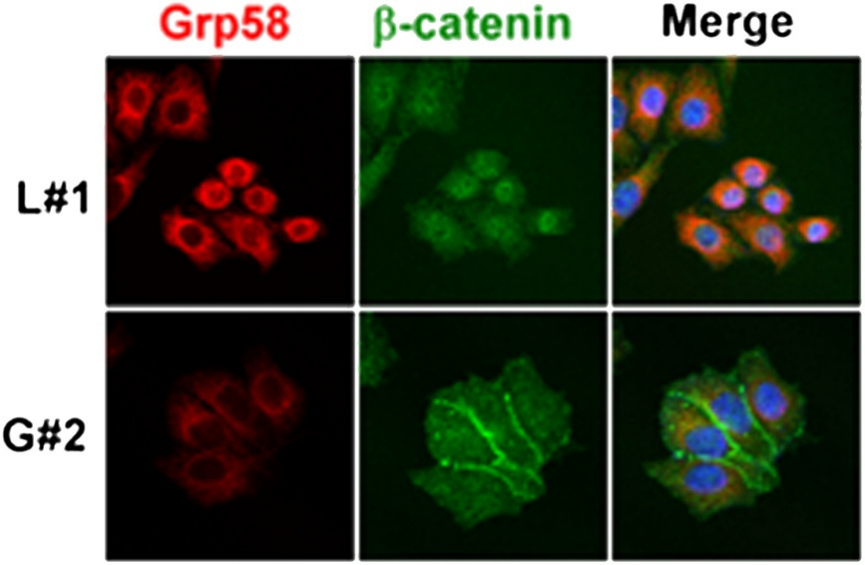
**Distribution of Grp58 and β-catenin in cervical cancer cells.** Grp58 and β-catenin immunoreactive signals of control (upper panel, L#1) and Grp58 knockdown (lower panel, G#2) cells were captured with confocal microscopy.

In general, β-catenin mainly localizes to the cytoplasmic membrane as a component of adherens junctions. Subcellular localization of β-catenin in stable HeLa cells was determined using IF staining, with images visualized using confocal microscopy. As shown in Figure 2, Grp58 was mainly located in the endoplasmic reticulum (ER), consistent with previous reports [7, 19]. Notably, broad membranous staining of β-catenin was observed in Grp58 knockdown, but not control, cells.

### β-catenin inhibits HeLa cell migration

**Figure 3:**
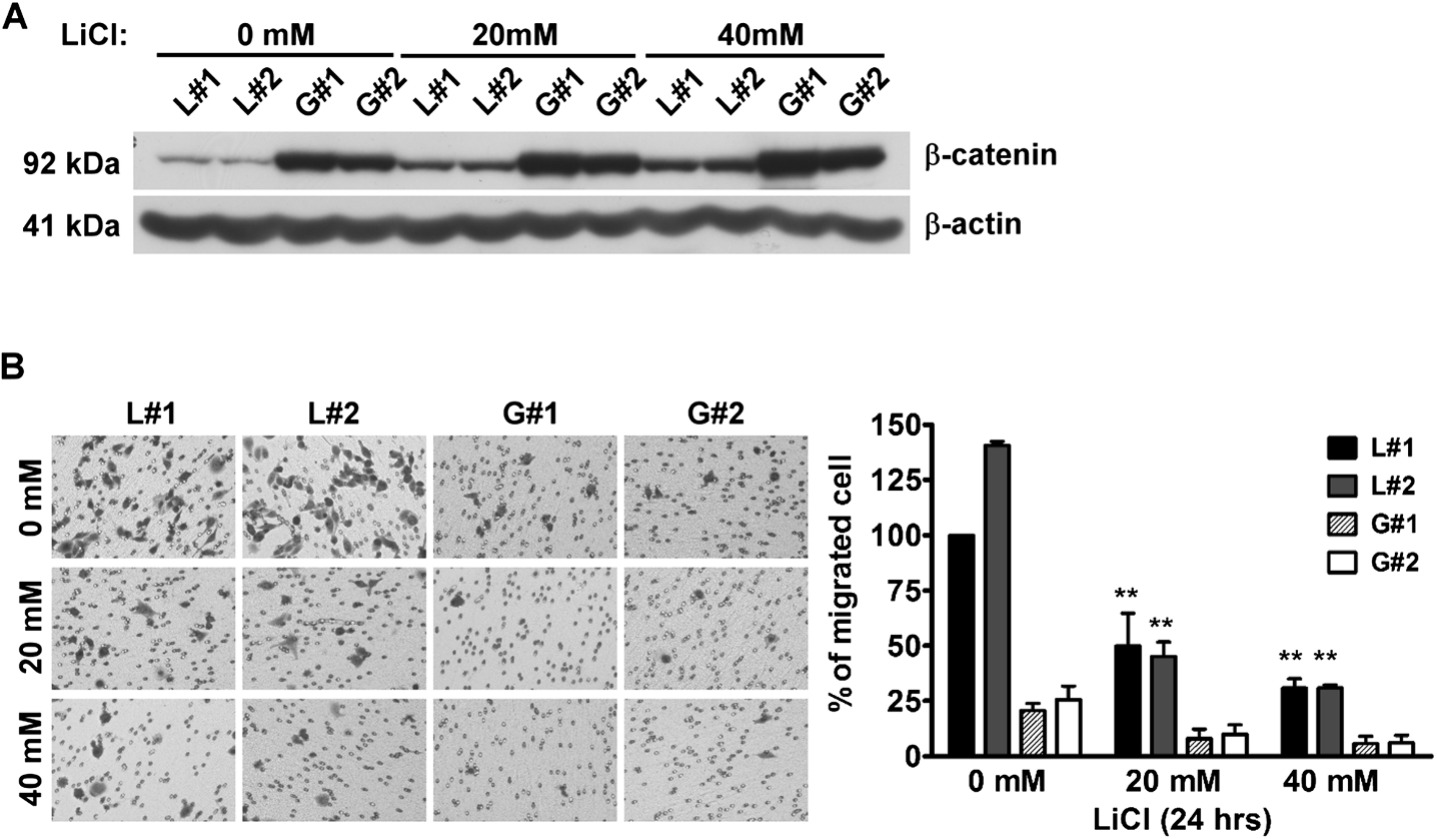
**Cell migration ability is inhibited by β-catenin. (A)** Western blot was used to determine β-catenin protein levels after treatment of cells with or without LiCl (20 and 40 mM) for 24 h. **(B)** The transwell assay was used to determine the migration abilities of cells treated with LiCl. Left panel, images of traversed cells; Right panel, Quantitative results of the transwell assay; **, *P* < 0.01.

To explore the impact of stable accumulated β-catenin on cell migration, the GSK3 inhibitor, LiCl, was used to suppress β-catenin protein degradation. LiCl enhanced β-catenin protein levels in a dose-dependent manner (Figure 3A). The migration abilities of cells treated with LiCl were further determined. As shown in Figure 3B, the migration abilities of L#1 and L#2 cells were significantly inhibited, along with β-catenin accumulation, in the presence of LiCl (Figure 3B). Consistent results were obtained with the Transwell invasion assay (Ref. [4] and Additional file 3: Figure S2A). The results indicate that β-catenin induced by Grp58 knockdown or LiCl treatment suppresses the migration ability of HeLa cells.

### Inverse expression patterns of Grp58 and β-catenin are observed in cervical cancer

**Figure 4:**
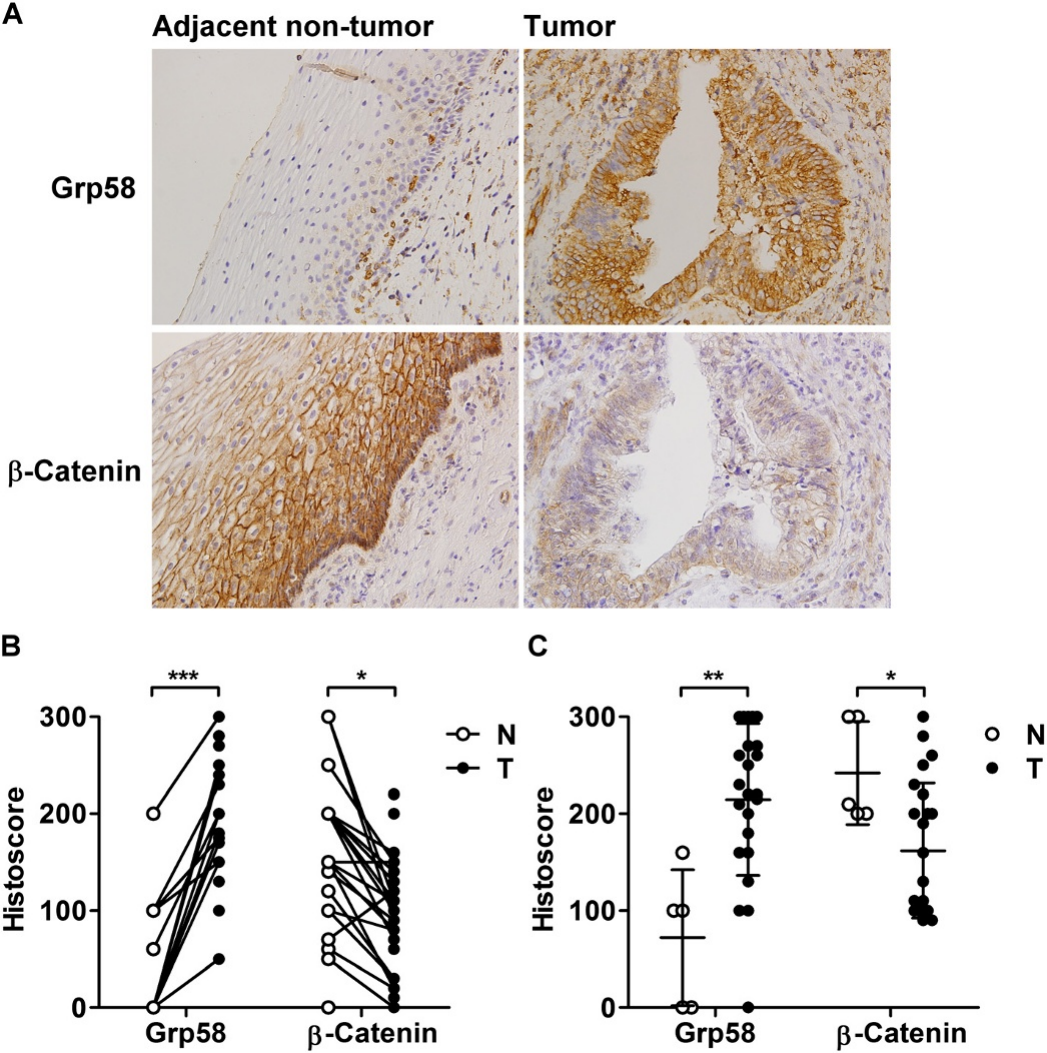
**Grp58 and β-catenin expression patterns in cervical AD. (A)** IHC staining for Grp58 and β-catenin was performed in 34 cervical AD patients. **(B)** The scatter plot shows Grp58 and β-catenin histoscores of 34 AD patients. The matched paired samples were linked with lines. **(C)** Expression of Grp58 and β-catenin was determined by performing IHC with commercial TMA. Among all 228 cases, 5 non-tumor epithelium tissues and 23 AD tissues in stage I to II tumors were scored. N, non-tumor tissue; T, tumor tissue; *, *P* < 0.05; **, *P* < 0.01; ***, *P* < 0.001.

Loss of membranous β-catenin is a common feature of various tumors, including cervical AD [20, 21]. Thus, expression patterns of β-catenin in our study population were determined. Intense β-catenin membranous staining was observed in adjacent normal epithelium, and most AD samples showed incomplete membranous staining (Figure 4A). As shown in Figure 4B, the β-catenin mean histoscore (Q, mean ± standard deviation) of adjacent normal tissues (149.5 ± 83.2) was significantly higher than that of tumor tissues (92.1 ± 62.3; Mann–Whitney test, *P* = 0.014). Conversely, the Grp58 level was significantly higher in tumors (186.2 ± 60) than adjacent normal tissues (29.5 ± 56.3; *P* < 0.001). In paired samples (Figure 4B, linked with lines), increased Grp58 histoscores were observed in all tumor tissues, compared with non-tumor regions, and decreased β-catenin histoscores recorded in 82.6 % tumor tissues. An inverse trend was evident in the expression of Grp58 and β-catenin. IHC staining for Grp58 and β-catenin was additionally applied to a commercial tissue microarray (TMA; CXC2281, Pantomics Inc., Hilltop Drive, CA). Grp58 was significantly overexpressed in AD (214.6 ± 78.4), compared with non-tumor epithelium (72 ± 70.1; *P* = 0.005), while β-catenin was significantly downregulated in AD (Q of AD and non-tumor epithelium were 161.7 ± 69.8 and 242 ± 53.1, respectively; *P* = 0.035; Figure 4C). This result was consistent with findings for our study population. The inverse expression trend of Grp58 and β-catenin was further validated in another cohort (Figure 4C).

## Discussion

β-Catenin is a protein with ambivalent functions. It serves as an adherent molecule to maintain epithelial phenotype of cell thereby inhibiting cell invasiveness. On the other hand, it is found in the nucleus together with LEF-1 transcription factor to drive a variety of target genes, such as epithelial-mesemchymal transition regulated genes, thereby enhances cell invasiveness [22, 23]. The present study of Grp58 regulation of cell invasiveness in cervical AD demonstrated that β-catenin is stabilized thereby accumulates in the membrane in Grp58 knockdown HeLa cells, thereby inhibiting migration ability. To our knowledge, this is the first study to provide evidence that Grp58 regulates WNT signaling. In our microarray experiment, several genes downstream of the WNT canonical pathway were identified. However this phenomenon appears to reflect a nuclear rather than a plasma membrane adherent function of β-catenin. Indeed, membrane-targeted β-catenin has been shown to increase the concentration of cytosolic β-catenin, which is necessary for transduction signals to the nucleus [24]. Nuclear β-catenin is thought to play an oncogenic role in tumorigenesis. However, earlier studies suggest that some potent invasion-promoting genes, such as S100A4 and NEDD9, are inhibited by the WNT canonical pathway [25]. Shtutman *et al.* demonstrated that induction of progressive multifocal leukoencephalophthy by β-catenin suppresses the tumorigenicity of renal carcinoma cells [26]. Microarray analysis led to the identification of S100A4 as a downregulated gene in the current study. The S100A4 protein level was verified using Western blot analysis (Additional file 2: Figure S1B). Decreased S100A4 expression may result in attenuation of migration and invasion abilities. However, restoration of S100A4 expression was not sufficient to rescue the migration and invasion phenotype (data not shown). Identification of the key molecules involved in Grp58-β-catenin-mediated regulation of cell invasiveness is thus of considerable interest.

Abnormal β-catenin expression, observed as loss of or reduced membranous staining, is a common feature of cervical cancer [21, 27], and alterations in β-catenin-related cell adherence are thought to be involved in cervical carcinoma pathogenesis [20]. In our study, all β-catenin positive staining cases shown a membranous fashion and nuclear staining of β-catenin was not observed in any cases. This result is identical with previous studies in cervical AD [20, 21]. β-Catenin function as a transcription activator may prefer to be an adhesion molecule in early stage cervical AD. Therefore, the accumulated β-catenin induced by Grp58 depletion or LiCl appears to play the adherent role and inhibited HeLa cell invasiveness. In our study population, membranous β-catenin was significantly decreased in a large proportion of AD tissues, compared to adjacent normal epithelium, which served as the normal control since the adjacent normal columnar epithelium was rarely observed on the tissue slide. Conversely, Grp58 was overexpressed in AD. We observed inverse expression patterns of Grp58 and β-catenin in our clinical specimens as well as a commercial TMA. In the cell-based study, knockdown of Grp58 expression resulted in accumulation of β-catenin around the plasma membrane of cells. Based on these results, we speculated that Grp58 acts as a regulator of β-catenin protein distribution and stability in cancer cells.

A previous study by our group demonstrated that Grp58 serves as an independent factor for cervical AD but not SCC [4]. Accordingly, we investigated the role of Grp58 in the cervical AD cell line HeLa. A migration assay was additionally performed with Caski and C33A, two SCC cell lines with Grp58 knockdown, as well as control cells without Grp58 knockdown. Migration abilities were moderately affected in the Grp58 knockdown cell lines, compared to control cells (Additional file 4: Figure S3). The regulatory effect of Grp58 on cell migration may thus be more significant in AD than SCC.

One of the most widely studied functions of Grp58 is its role in the immune system. Grp58 participation in MHC class I antigen presentation is well documented [28]. Consistent with this, the “Antigen presentation by MHC class I” pathway was the third most significantly enriched in our microarray analysis. Alterations in adaptive immune responses have been reported in cervical cancer [29]. Additionally, Cromme *et al.* demonstrated that MHC class I is downregulated in metastases from cervical carcinoma compared with the primary tumors [30]. Therefore, we speculated that Grp58 regulates the adaptive immune response to augment cancer invasion. Granzyme A (GZMA) signaling was the most significantly affected pathway in our enrichment analysis which is mainly attributed to differential expression of SET complex, the central component in the GZMA pathway (Table 1 and ref. [4]). Four members of the SET complex, including SET, high mobility group box 2 (HMGB2), APEX nuclease 1 (APEX1) and acidic leucine-rich nuclear phosphoprotein 32 family member A (ANP32A), are affected by Grp58 silencing [4]. SET complex responses to GZMA and oxidative stress represents in tumors to regulate cell apoptosis [31]. Downregulation of homeostatic ER stress responses via knockdown of Grp58 expression appears to enhance apoptosis induced by oxidative stress-inducing drugs [32]. Accordingly, knockdown of Grp58 expression may disrupt ER homeostasis, resulting in accumulation of oxidative stress and changes in the status of the SET complex. The detailed molecular mechanism underlying Grp58-mediated apoptosis is unclear. It would be of interest to determine whether Grp58 regulates apoptosis through the SET complex and associated proteins that participate in cervical cancer progression and drug resistance.

## Conclusions

Patients with cervical AD are generally considered to have a poorer prognosis than those with SCC [33]. However, knowledge of the natural history and optimal management of cervical AD is limited. Early detection, prognosis and treatment strategies specific to AD should be explored in future studies. Previously we identified Grp58 as an independent prognostic marker for cervical AD [4]. Here we have demonstrated that Grp58 appears to regulate WNT signaling by targeting β-catenin to augment cancer invasion. Regulation of the immune response and free radical homeostasis are possible mechanisms underlying cervical cancer progression. Further research is warranted to determine the detailed mechanism of Grp58 action in cervical cancer progression.

## Electronic supplementary material

Additional file 1: **Supplementary methods and figures.** (DOCX 23 KB)

Additional file 2: Figure S1: Verification of the expression of WNT signaling pathway-related genes. (TIFF 12 MB)

Additional file 3: Figure S2: Invasion and proliferation abilities of stable cells. (TIFF 7 MB)

Additional file 4: Figure S3: Migration abilities of Grp58 knockdown Caski and C33A cells. (TIFF 6 MB)
