# Supplementary material for: Glucose-regulated protein 58 modulates β-catenin protein stability in a cervical adenocarcinoma cell line
Source: BMC Cancer. 2014 Aug 1;14:555. doi: 10.1186/1471-2407-14-555 (PMC4129111; doi:10.1186/1471-2407-14-555)
Supplement: Supplementary file 1 — Additional file 1: Supplementary methods and figures. (DOCX 23 KB) [file 12885_2013_4741_MOESM1_ESM.docx]

**Supplementary methods and figures**

**Methods**

**Primers**

The sequences of Primers used in qRT-PCR were CSNK1E: 5’-Agg TgA gTA TgA ggC TgC AC-3’, 5’-AgA Tgg TCA AAT ggC ACA CT-3’; DVL-1: 5’-CCA Agg CCT ATA CAg Tgg Tg-3’, 5’-CACATgATgTCCACgAAgAA-3’; FZD10: 5’-gTg CAg CCg Tag gTT AAA gA-3’, 5’-CTC ATA TTT CCC gTg gTg Ag-3’; L1CAM: 5’-TCg CCA CAg TAT gTC AgC TAC A-3’, 5’-ggC CTg TgC CAT Tgg TCT T-3’; LAMB3: 5’-CCT CAC AAC TAC TAC AgT CAC CgA gTA-3’, 5’-CAg AgA gAC Agg gTT CAC ATC ATT C-3’; LEF1: 5’-ACA ACA Agg gAC CCT CCT AC-3, 5’-ggATggTggAgAAAgAgAT-3’; Myc: 5’-Agg AgA CAT ggT gAA CCA gA-3’, 5’-CTT CTC TgA gAC gAg CTT gg-3’; S100A4: 5’-AgA Agg CCC Tgg ATg TgA Tg-3’, 5’-CTg ACT TgT TgA gCT TgA ACT TgT C-3’.

**Western Blot**

The antibodies used in Western Blot were as follow: CD44 primary antibody (GeneTex, Inc., Hsinchu, Taiwan, R.O.C.), 1:1000 dilution; L1CAM primary antibody (abcam, Interlab Co., Ltd, Taipei, Taiwan, R.O.C.), 1:2000 dilution; Myc (GeneTex), 1:1000 dilution; S100A4 (abcam), 1:1000 dilution.

**TCF-responsive reporter assay**

TCF-responsive reporter assay was used to determined β-catenin-TCF transcription activity and was performed as previous described [[1](#_ENREF_1)].

**Proliferation assay**

The cells were harvested by trypsinization and 1x10^4^ cells were seeded in 60 mm culture dish. The cells were counted with hemocytometer at day1, 3, 5 and 7.

**Figures**

**Figure S1 - Wnt signaling pathway is regulated upon knockdown of Grp58 expression**

Cohort of Wnt signaling-related genes identified in our previous microarray analysis by comparing control and Grp58 stable knockdown cells were verified by performing qRT-PCR (A) and Western Blot (B). These results were consistent with that of DNA microarray. (C) The β-catenin-TCF transactivation activities of stable cells were determined by performing TCF-responsive reporter assay. *, *P*<0.05; **, *P*<0.01; ***, *P*<0.001

**Figure S2 - The invasion and proliferation ability of stable cells**

A, Transwell invasion assay was performed to analysis the invasiveness of control and Grp58-knockdown cells treated with or without LiCl. B, Proliferation assay was performed to determine the proliferation activities of control (L#1 and L#2) and Grp58-knockdown stable cell lines (G#1 and G#2). No significant difference in proliferation was observed among these stable cells. **, *P*<0.01; ***, *P*<0.001

**Figure S3 - Migration abilities of Grp58 depletion Caski and C33A cell lines**

A, Grp58 protein levels of three cervical cancer cell lines stably transfected with control or Grp58 shRNA were determined using Western blot. These three lines express comparable high endogenous Grp58. B, migration abilities of stably control and Grp58 knockdown Caski and C33A cells were determined using Transwell migration assay. *, *P* < 0.05; student’s *t*-test

**References**

1. Loh YN, Hedditch EL, Baker LA, Jary E, Ward RL, Ford CE: **The Wnt signalling pathway is upregulated in an in vitro model of acquired tamoxifen resistant breast cancer.** *BMC Cancer* 2013, **13:**174.
