# Supplementary figures and images for: Glucose-regulated protein 58 modulates β-catenin protein stability in a cervical adenocarcinoma cell line
Source: BMC Cancer. 2014 Aug 1;14:555. doi: 10.1186/1471-2407-14-555 (PMC4129111; doi:10.1186/1471-2407-14-555)

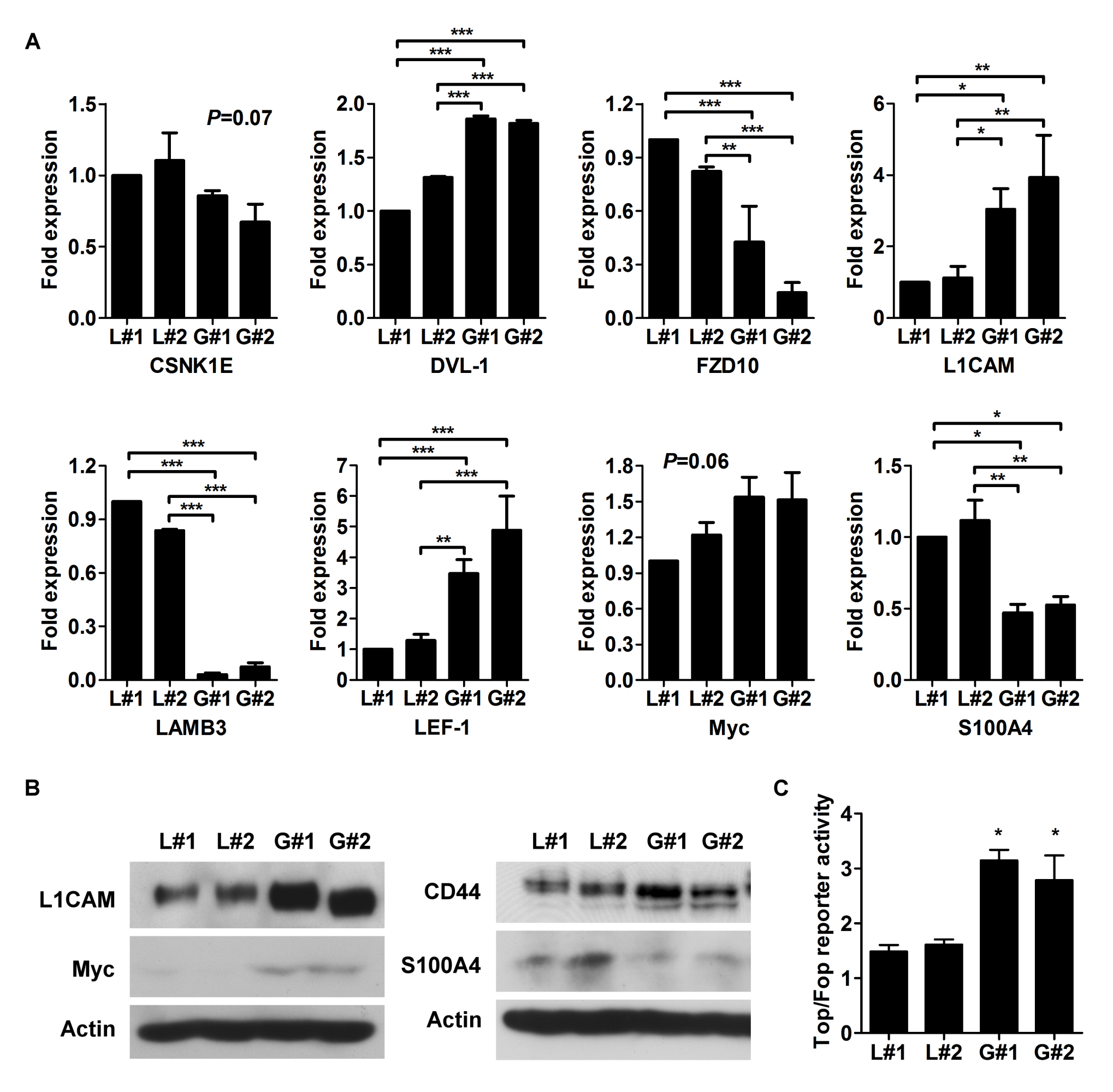

Supplement: Supplementary file 2 — Additional file 2: Figure S1: Verification of the expression of WNT signaling pathway-related genes. (TIFF 12 MB) [file 12885_2013_4741_MOESM2_ESM.tiff]

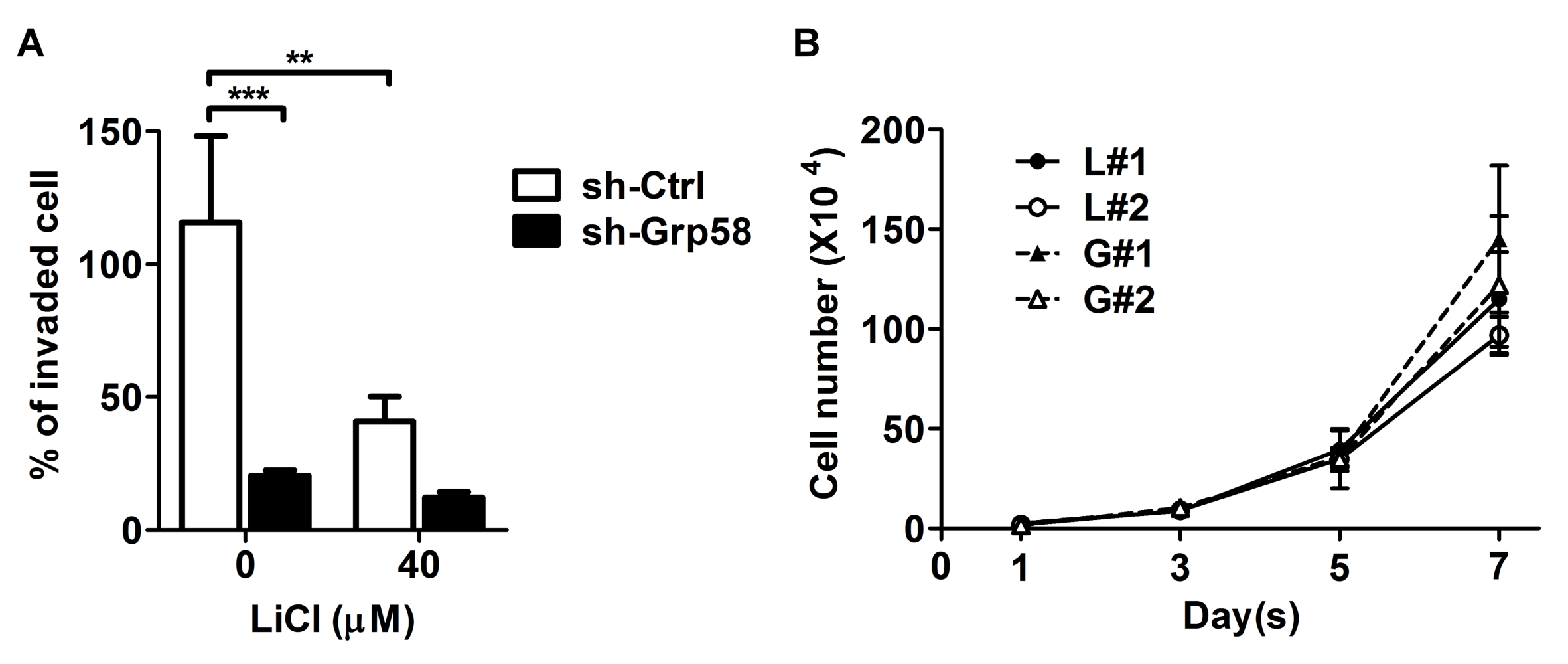

Supplement: Supplementary file 3 — Additional file 3: Figure S2: Invasion and proliferation abilities of stable cells. (TIFF 7 MB) [file 12885_2013_4741_MOESM3_ESM.tiff]

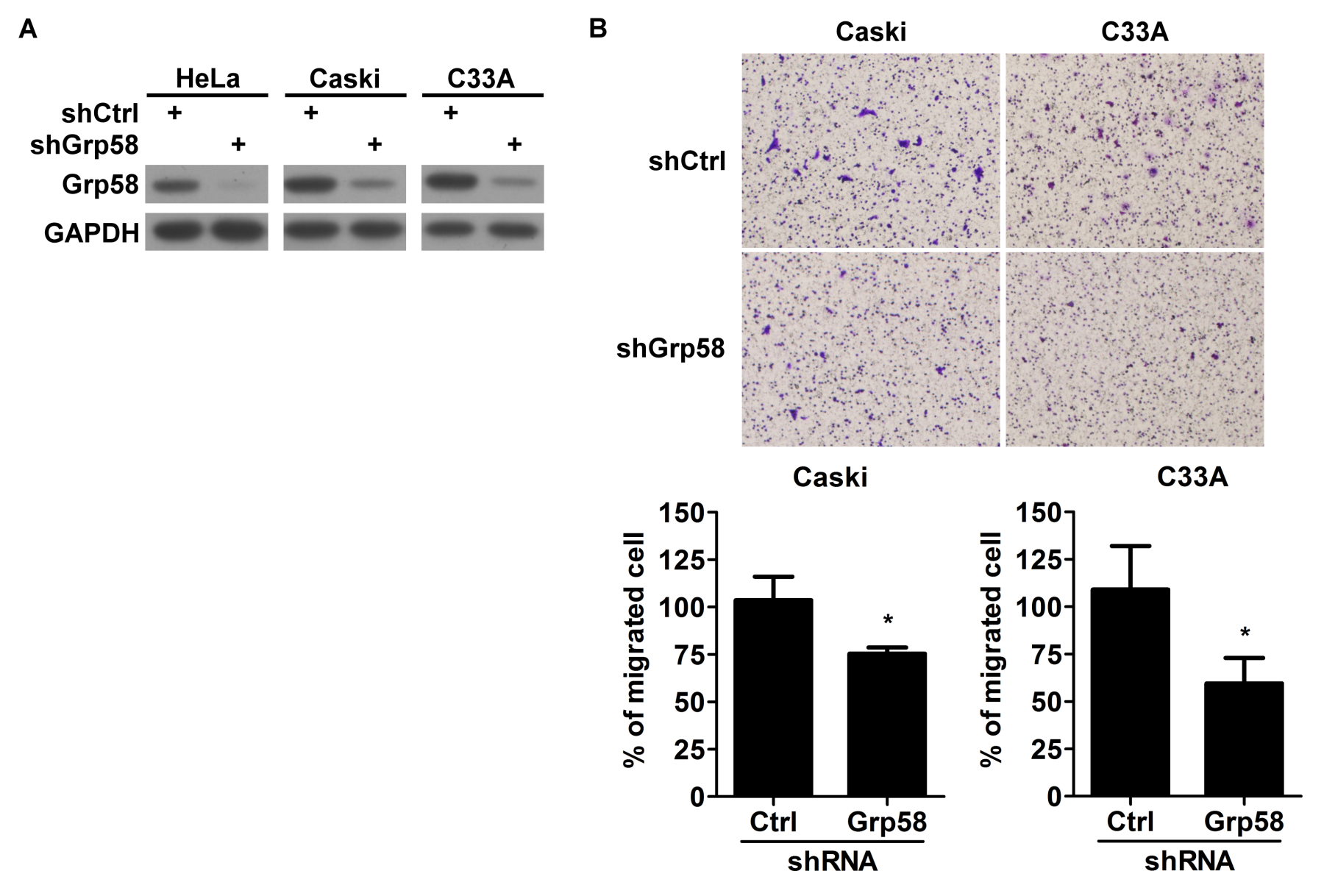

Supplement: Supplementary file 4 — Additional file 4: Figure S3: Migration abilities of Grp58 knockdown Caski and C33A cells. (TIFF 6 MB) [file 12885_2013_4741_MOESM4_ESM.tiff]
